# An Incidental Finding of Takotsubo Cardiomyopathy in a Trauma Case

**DOI:** 10.7759/cureus.35106

**Published:** 2023-02-17

**Authors:** Talha Shabbir, Sarala Kal, Saloni Gupta, Rachel Hunsucker, Danish Siddiqui

**Affiliations:** 1 Research, California University of Science and Medicine, Colton, USA; 2 Internal Medicine, Arrowhead Regional Medical Center, Colton, USA; 3 Internal Medicine, California University of Science and Medicine, Colton, USA; 4 Cardiology, Loma Linda University Medical Center, Loma Linda, USA

**Keywords:** takotsubo cardiomyopathy (tc), transesophageal echocardiogram, catheterization, echocardiogram, general trauma surgery, interventional cardiology

## Abstract

Takotsubo cardiomyopathy (TC) is a transient condition characterized by left ventricular wall motion abnormalities, ventricular systolic dysfunction, and apical ballooning. When initially presented, the pathology is often erroneously attributed to acute coronary syndrome (ACS) or acute-onset heart failure due to similar symptoms and electrocardiogram (ECG) findings. However, upon further review of imaging, coronary arteries are often void of disease. The highest prevalence of Takotsubo cardiomyopathy is noted in elderly, postmenopausal women who recently experienced an emotionally or physically triggering event. Although the true underlying pathophysiology of Takotsubo cardiomyopathy remains poorly elucidated, a few leading concepts suggest that stress-induced sympathetic responses may lead to catecholamine-induced cardiotoxicity. Other ideologies implicate poor coronary perfusion, neurogenic myocardial stunning, and coronary artery vasospasms. As features of TC are transient, it has an excellent prognosis, and patients see improvement in ventricular function and symptoms within weeks after the initiation of therapy. In this paper, we discuss a case of TC noted incidentally on imaging in a middle-aged female presenting with encephalopathy after a motor vehicle accident.

## Introduction

First discussed by Japanese cardiovascular physicians in 1990, Takotsubo cardiomyopathy (TC) is a transient condition characterized by left ventricular wall motion abnormalities, ventricular systolic dysfunction, and apical ballooning [1-9]. Additional hallmark findings include transient dyskinesis of the middle or apical cardiac segments and hyperkinesis of the basal cardiac segments [4-8]. Takotsubo, or “octopus pot” in Japanese, refers to the pathological shape of the ventricles noted on imaging during the end of systole [1-5,9-11].

The condition is relatively rare, with an incidence of 1%-2% in patients presenting with symptoms and findings consistent with acute coronary syndrome (ACS) [2,6-8,10,11]. In the United States, TC is even less widespread, with a diagnosis occurring in about 0.02% of all hospitalizations [12]. On the other hand, hospital data in areas suffering from natural disasters have shown a higher incidence of TC than those without an underlying stressor [2]. The highest prevalence is noted in elderly, postmenopausal women who recently experienced an emotionally or physically triggering event [1,2,9,11].

Fortunately, as features of TC are transient, patients typically have a favorable prognosis and often improve with supportive care and heart failure therapy [1,3,4,8,13]. In this paper, we discuss a case of TC noted incidentally on imaging in a middle-aged female presenting with encephalopathy after a motor vehicle accident.

## Case presentation

A 36-year-old female with an unknown past medical history presented by air transport after a vehicle accident. In the field, the patient had a Glasgow Coma Scale (GCS) score of 5 and required intubation. On arrival, the patient presented with blunt chest trauma and encephalopathy. A primary inspection revealed a head laceration, forehead hematoma, and multiple abrasions to the head and lower extremities. Initial laboratory findings were significant for tachycardia and leukocytosis. The urine drug screen was positive for marijuana, and the patient’s blood alcohol concentration (BAC) was elevated at 0.131%. Computed tomography (CT) of the head without contrast revealed bilateral temporal subarachnoid hemorrhage with extra-axial hemorrhage around the anterior falx and scalp swelling in the frontal and left parietal regions. CT of the chest, abdomen, and pelvis with contrast noted bilateral pulmonary contusions and atelectasis. A repeat head CT indicated stabilization of the hemorrhage; however, the patient continued to display signs of confusion with amnesia to the event and to self.

**Figure 1:**
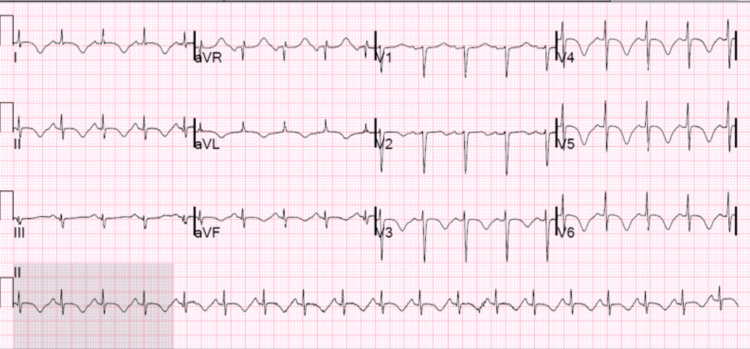
ECG showing diffuse T-wave inversions more prominent in the precordial leads ECG: electrocardiogram

**Figure 2:**
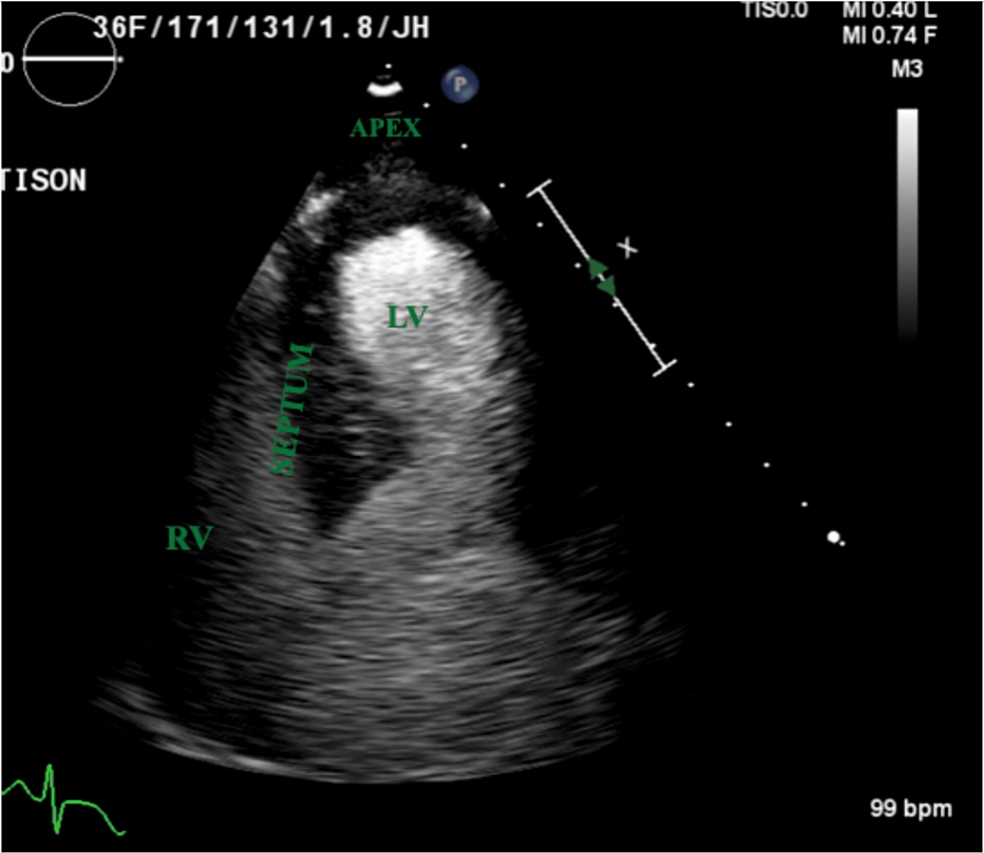
Echocardiogram showing apical ballooning in systole LV: left ventricle, RV: right ventricle

**Figure 3:**
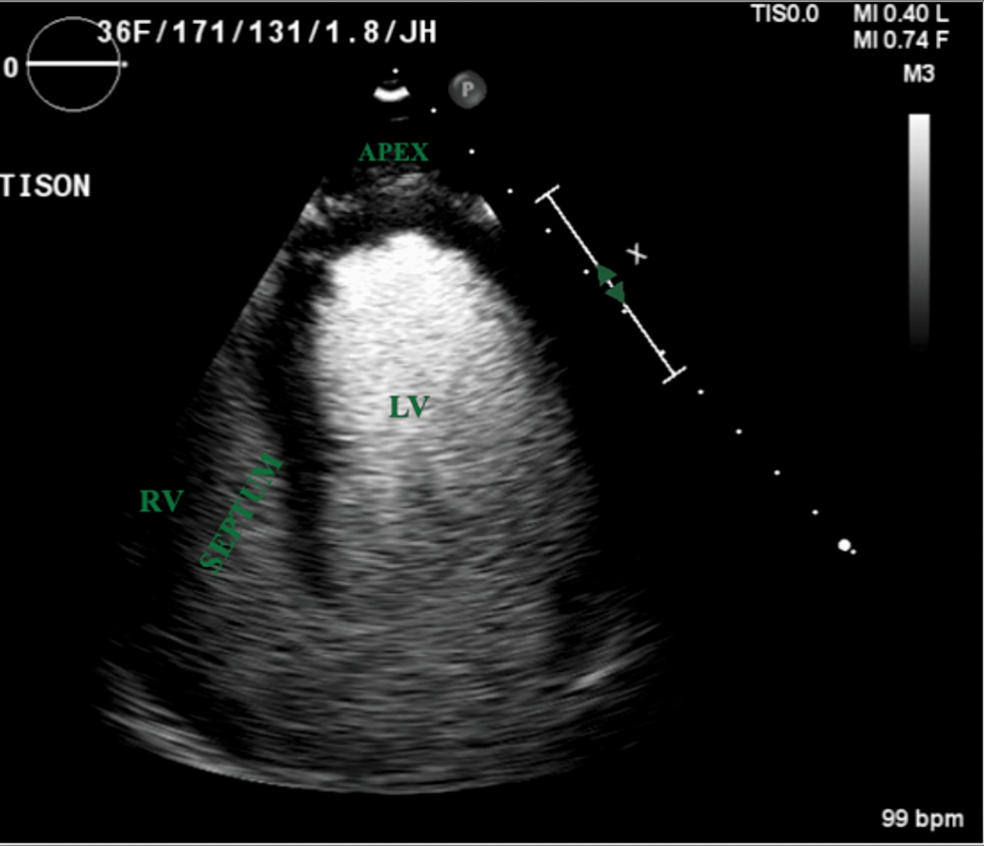
Echocardiogram showing apical ballooning in diastole LV: left ventricle, RV: right ventricle

A few days later, the patient developed a prolonged QT interval and a short run of ventricular tachycardia. ECG was consistent with diffuse T-wave inversions (Figure 1). Laboratory findings showed unremarkable cardiac enzymes and creatinine kinase downtrending from admission. Echocardiogram revealed an ejection fraction of 40% and a severely hypokinetic apex with a hyperkinetic basal segment consistent with Takotsubo cardiomyopathy (Figure 2 and Figure 3). A coronary CT angiogram revealed no evidence of coronary stenosis or plaque with a calcium score of zero. The patient was treated with metoprolol tartrate 25 milligrams twice daily and lisinopril 2.5 milligrams daily. The patient remained altered and confused with a GCS score of 14 but began to worsen a few days later. Repeat CT head noted a decreased subarachnoid and subdural hematoma compared to the prior. The constellation of clinical symptoms, coupled with the patient’s recent history of a motor vehicle accident, ruled up a diagnosis of traumatic brain injury (TBI) with suspected diffuse axonal injury compounding multifocal traumatic intracranial hemorrhage. A subsequent electroencephalogram (EEG) was unremarkable.

Throughout the following days, the patient remained somnolent with fluctuating mentation. A repeat echocardiogram completed showed improvement in cardiomyopathy, with an improved ejection fraction estimated at 60%. Additional conversations with the family revealed that the patient was going through a divorce and had complications regarding custody of her children. This inciting stress along with the traumatic vehicle accident was thought to have been the emotional stressors triggering her TC. The patient was discharged with lisinopril and outpatient follow-up.

## Discussion

Within the last decade, TC has become increasingly well-recognized and documented [2]. Although a concrete diagnostic outline has yet to be established, the Mayo Clinic highlighted critical features of the syndrome in 2004 [1-5,7-11]. The criteria include the following: (1) the presence of transient hypokinesis, akinesis, or dyskinesis in the left ventricular mid-segments with or without apical involvement; regional wall motion abnormalities that extend beyond a single epicardial vascular distribution; and frequently, but not always, a stressful trigger; (2) the absence of obstructive coronary disease or angiographic evidence of acute plaque rupture; (3) new ECG abnormalities (ST-segment elevation and/or T-wave inversion) or modest elevation in cardiac troponin; and (4) the absence of pheochromocytoma and myocarditis [1-5,7-11]. The patient in this report satisfied the Mayo Clinic diagnostic criteria after telemetry noted electrical abnormalities, an echocardiogram showed a severely hypokinetic apex with a hyperkinetic basal segment, and an additional workup was void of alternative pathologies.

On initial presentation, TC is often erroneously attributed to acute coronary syndrome (ACS) or acute-onset heart failure due to similar symptoms and electrocardiogram findings [4,5,8-11,13]. However, upon further review of imaging studies, such as coronary angiography, coronary arteries are often void of significant coronary artery disease [1,8]. In this case, the patient had altered mental status and did not present signs and symptoms of ACS. However, telemetry readings during hospitalization showed a prolonged QT interval, T-wave inversions, and a short run of ventricular tachycardia. As such, an echocardiogram followed by a CT angiogram was ordered, which confirmed the presence of TC while ruling out ACS. Coronary catheterization was not considered necessary at this time given the young age and normal biomarkers of the patient and the overall clinical picture consistent with TC.

Once diagnosed with TC, acute treatment typically involves the management of patient symptoms via supportive care and treatment of complications [1,3,4,8,13]. Subsequent treatment modalities are dependent on patient status. Hemodynamically stable patients are treated with systolic heart failure protocols, including low-dose beta-blockers, angiotensin-converting enzyme (ACE) inhibitors, and diuretics [10]. Similarly, the patient, in this case, was started on metoprolol tartrate for heart rate control and lisinopril for cardioprotective effects.

Hemodynamically unstable patients may require cardiopulmonary support and treatment with inotropes, an intra-aortic balloon pump, or other therapies [1,3,7,10]. The use of inotropes remains controversial due to the side effect profiles and possible aggravation of cardiomyopathy or left ventricular outflow tract obstruction from increased catecholamines [5,7,8,10,13]. Lastly, treatment for underlying stressors or comorbidities can be initiated to reduce the disease burden [1,7].

TC has an excellent prognosis, and patients see improvements in ventricular function and symptoms within weeks [1-5,7,8,10]. One study estimated the in-hospital mortality rate for TC patients at 4%-5% [11]. Nevertheless, when diagnosed and treated early, TC remains medically manageable with favorable outcomes [1-5,7,8,10]. Likewise, the patient in this report had marked improvement on her repeat echocardiogram with an improved ejection fraction estimated at 60%.

There are many theories behind the etiology of stress causing cardiomyopathy. One hypothesis suggests that an emotionally triggering event may result in catecholamine-induced cardiotoxicity. Other studies have also implicated neurogenic involvement in myogenic stunning, leading to reversible cardiomyopathy [8,9]. That theory suggests that acute neurologic injuries such as subarachnoid hemorrhage or stroke can lead to subsequent dysfunction, particularly in the nerves of the coronary microvasculature, resulting in myocardial stunning [8,9]. As such, it is possible that the patient’s accident, which resulted in bilateral temporal subarachnoid hemorrhage and subsequent traumatic brain injury, may have been the inciting factor leading to the development of TC.

## Conclusions

This case report discusses a patient who was incidentally found to have Takotsubo cardiomyopathy after a traumatic car accident. Her hospital course highlights the acute findings and subsequent resolution with timely diagnosis and appropriate treatment. Due to its many alternative manifestations, it is important that clinicians have a high suspicion for Takotsubo cardiomyopathy and understand the various presentations, methods of diagnosis, and treatment regimens.
